# 441. Prior SARS-CoV-2 Diagnosis is a Risk Factor for Herpes Zoster in Younger Adults: A Nested Case-control Study in the EPICC Cohort

**DOI:** 10.1093/ofid/ofad500.511

**Published:** 2023-11-27

**Authors:** Kat Schmidt, Stephanie A Richard, Catherine Berjohn, Tahaniyat Lalani, Alfred Smith, Rupal Mody, David Lindholm, Nikhil Huprikar, David Saunders, Rhonda Colombo, Milissa Jones, Ryan Flanagan, Evan Ewers, David Chang, Christina Schofield, Margaret Sanchez Edwards, Carlos Maldonado, Katrin Mende, Ann Scher, Jennifer Rusiecki, Celia Byrne, Andrew Wyatt, Mark P Simons, David R Tribble, David R Tribble, Robert O’Connell, Brian Agan, Timothy Burgess, Simon Pollett, Anuradha Ganesan

**Affiliations:** Infectious Disease Clinical Research Program, USUHS, Arlington, Virginia; Infectious Disease Clinical Research Program, Department of Preventive Medicine and Biostatistics, Uniformed Services University of the Health Sciences, Bethesda, MD, USA, Bethesda, Maryland; Naval Medical Center San Diego, San Diego, California; Naval Medical Center Portsmouth, Portsmouth, Virginia; Naval Medical Center, Portsmouth, Virginia; William Beaumont Army Medical Center, El Paso, Texas; Department of Medicine, Uniformed Services University of the Health Sciences; Brooke Army Medical Center, San Antonio, Texas; Walter Reed National Military Medical Center, Bethesda, Maryland; Uniformed Services University of the Health Sciences, Bethesda, MD, USA, Bethesda, Maryland; Infectious Disease Clinical Research Program, USUHS, Arlington, Virginia; Uniformed Services University of the Health Sciences, Bethesda, Maryland; Tripler Army Medical Center, Honolulu, Hawaii; Fort Belvoir Community Hospital, Fort Belvoir, Virginia; Fort Belvoir Community Hospital, Fort Belvoir, Virginia; Madigan Army Medical Center, Tacoma, Washington; Infectious Disease Clinical Research Program, Department of Preventive Medicine and Biostatistics, Uniformed Services University of the Health SciencesHenry M. Jackson Foundation for the Advancement of Military Medicine, Bethesda, Maryland; Womack Army Medical Center, Fort Bragg, North Carolina; Brooke Army Medical Center, San Antonio, Texas; Uniformed Services University of the Health Sciences, Bethesda, Maryland; Uniformed Services University of the Health Sciences, Bethesda, Maryland; Uniformed Services University of the Health Sciences, Bethesda, Maryland; Landstuhl Regional Medical Center, Landstuhl, Rheinland-Pfalz, Germany; Infectious Disease Clinical Research Program, Department of Preventive Medicine and Biostatistics, Uniformed Services University of the Health Sciences, Bethesda, MD, USA, Bethesda, Maryland; Uniformed Services University of the Health Sciences, Bethesda, Maryland; Uniformed Services University of the Health Sciences, Bethesda, Maryland; Infectious Disease Clinical Research Program, USUHS, Arlington, Virginia; Infectious Disease Clinical Research Program, Department of Preventive Medicine and Biostatistics, Uniformed Services University of the Health Sciences, Bethesda, MD, USA, Bethesda, Maryland; Infectious Disease Clinical Research Program, Department of Preventive Medicine and Biostatistics, Uniformed Services University of the Health Sciences, Bethesda, MD, USA, Bethesda, Maryland; Infectious Disease Clinical Research Program, Department of Preventive Medicine and Biostatistics, Uniformed Services University of the Health Sciences, Bethesda, MD, USA, Bethesda, Maryland; Infectious Disease Clinical Research Program, USUHS; Henry M. Jackson Foundation for the Advancement of Military Medicine Inc, Bethesda, Maryland

## Abstract

**Background:**

Prior studies suggest SARS-CoV-2 (SC2) infection and vaccination are risk factors for the development of Herpes Zoster (HZ). We performed a nested case-control study using data from the Epidemiology, Immunology and Clinical Characteristics of Emerging Infectious Diseases of Pandemic Potential (EPICC) cohort to evaluate the impact of SC2 exposure (positive SC2 test or COVID-19 vaccination) on the odds of HZ diagnosis.

**Methods:**

The EPICC cohort is comprised primarily of younger (median age 34.6 years) Military Health System (MHS) beneficiaries who were either tested for SC2 and/or received COVID-19 vaccination. MHS Data Repository (MDR) records were evaluated for HZ diagnosis. Information about SC2 diagnosis and COVID-19 vaccination was aggregated from participant survey data and MDR records. Cases were defined as adult participants with an HZ ICD-10 code after 3/1/2020. Controls were selected randomly and matched individually to cases on age, race, sex, and time period. We defined exposure as ≥ 1 SC2 positive test or COVID-19 vaccination 0-365 days before HZ diagnosis. We used conditional logistic regression to explore the association between SC2 diagnosis, or COVID-19 vaccination, and HZ diagnosis, adjusting for comorbidities and HZ vaccination.

**Results:**

Between 3/2020 and 12/2022, 7,394 were enrolled in the EPICC study, and a total of 94 HZ cases were identified. The median age at HZ diagnosis was 39 years (IQR: 33, 48). There was no difference between the median age of HZ cases with and without SC2 diagnosis (38.5 and 39.4 years respectively, p=0.96). Among HZ cases, 28 (30%) had ≥ 1 SC2 diagnosis in the year prior, compared to 12 controls (13%) (Table 1). Participants with a known history of SC2 diagnosis were more likely to be HZ cases (OR 2.7; 95% CI: 1.3, 5.8), adjusting for HZ vaccination and Charlson Comorbidity Index (Table 2). No significant association was found between COVID-19 vaccination and HZ (OR: 1.0; 95% CI: 0.4, 2.5). Sensitivity analyses (exposure 1-90 days prior to HZ diagnosis) found similar results (Table 2).

**Figure:**
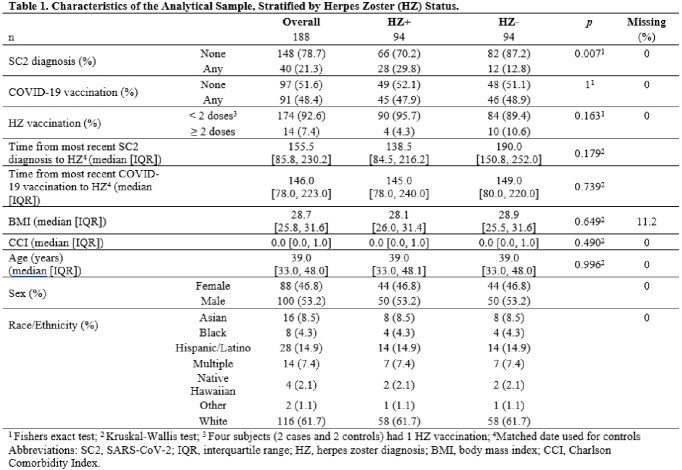

**Figure:**
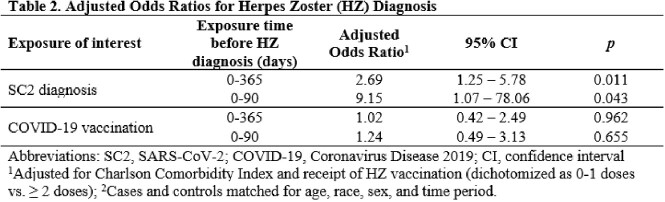

**Conclusion:**

In a cohort primarily comprised of younger individuals, SC2 diagnosis, but not COVID-19 vaccination, was associated with increased risk for HZ diagnosis.

**Disclosures:**

**Mark P. Simons, PhD**, AstraZeneca: The IDCRP and HJF were funded to conduct an unrelated phase III COVID-19 monoclonal antibody immunoprophylaxis trial as part of US Govt COVID Response **Timothy Burgess, MD, MPH**, AstraZeneca: The IDCRP and the Henry M. Jackson Foundation (HJF) were funded to conduct an unrelated phase III COVID-19 monoclonal antibody immunoprophylaxis trial **Simon Pollett, MBBS**, AstraZeneca: The IDCRP and the Henry M. Jackson Foundation (HJF) were funded to conduct an unrelated phase III COVID-19 monoclonal antibody immunoprophylaxis trial

